# Development of a 3-DOF Flexible Micro-Motion Platform Based on a New Compound Lever Amplification Mechanism

**DOI:** 10.3390/mi12060686

**Published:** 2021-06-11

**Authors:** Fangni Cui, Yangmin Li, Junnan Qian

**Affiliations:** 1 Tianjin Key Laboratory for Advanced Mechatronic System Design and Intelligent Control, Tianjin University of Technology, Tianjin 300384, China; fangnicui@163.com (F.C.); junnanqian@163.com (J.Q.); 2 Department of Industrial and Systems Engineering, The Hong Kong Polytechnic University, Hung Hom, Hong Kong 999077, China

**Keywords:** lever amplifying mechanism, flexure hinge, micromotion platform, finite element analysis

## Abstract

In this paper, a flexible micro-operation platform with three degrees of freedom, large stroke, and high precision is designed to meet the higher demands in the fields of biological engineering and medicine. The platform adopts a compound lever mechanism. The theoretical magnification of the mechanism is 9.627, the simulation magnification is 10.111, and the error is 5.02%. The platform uses a piezoelectric ceramic driver to increase the output stroke to obtain a larger movement space. The composite lever mechanism and new micro-operating platform are studied by theoretical calculation and finite element simulation. The results show that the new flexible micro-operating platform based on the composite lever mechanism has good motion decoupling and high precision.

## 1. Introduction

Flexible mechanisms based on flexure hinges are often used in biological cell manipulation, optical fiber alignment, and ultra-precision positioning platforms for scanning probe microscopes [1,2]. They are also increasingly used in many other aerospace and instrument fields, such as shape active control of aircraft [3], fluid control of servo valves [4], fatigue testing of microstructures [5], etc. Various multistage flexible mechanisms are increasingly designed for precision positioning platforms to achieve large workspaces and multiple degrees of freedom [6,7,8,9,10,11]. However, the output range of piezoelectric ceramics is very small, usually only a few microns to tens of microns. Therefore, in the precision operation requiring large stroke, the micro displacement amplification mechanism is usually selected to achieve the amplification and transmission of the output displacement of piezoelectric ceramics [12,13,14]. Common displacement amplification mechanisms based on a flexure hinge include lever amplification, Moonie-type [15], bridge-type amplification [16,17], Rainbow-type [18], and Scott–Russell [19,20,21,22,23]. The motion amplification mechanism, based on the lever principle, is simple in structure and convenient for processing [24]. However, due to the limitations of space, limited one-stage magnification, serious distortion, and large multi-stage magnification volume, two-stage lever amplification is usually adopted [25,26].

The new flexible micro-operation platform designed in this paper adopts a flexible mechanism combining the lever principle and a flexible hinge to magnify the drive displacement by 9 times and is verified by finite element simulation. In the remainder of this paper, Section 2 describes the amplification mechanism in detail. Section 3 describes finite element simulation analysis, Section 4 kinematics analysis, and Section 5 summarizes some conclusions.

## 2. Mechanism Description

**Figure 1:**
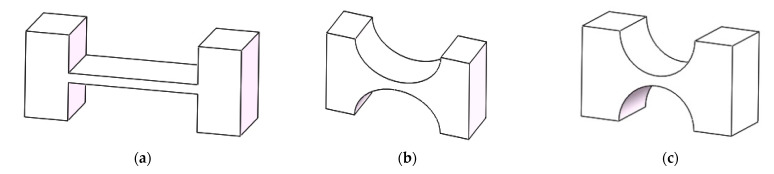
Different notched flexure hinges: (**a**) right-angle incision; (**b**) elliptical incision; (**c**) circular incision.

**Figure 2:**
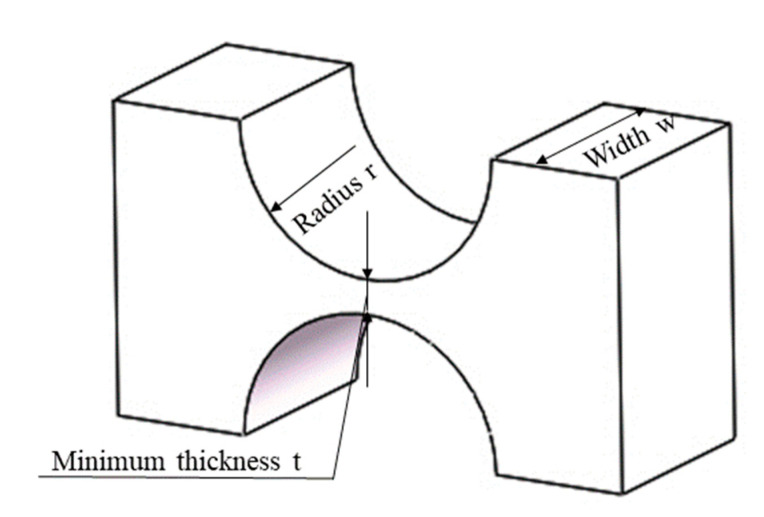
Straight round flexure hinge.

**Figure 3:**
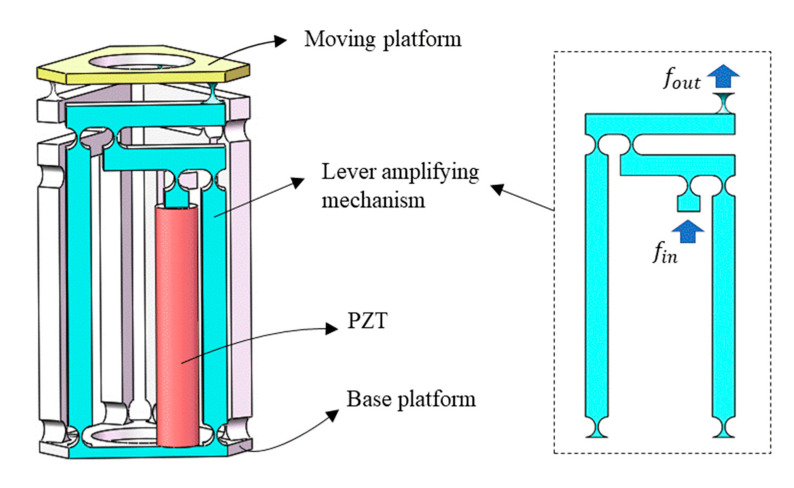
Model of a 3-DOF flexible micro-motion platform.

Generally speaking, flexible hinge incision types include a right-angle incision, elliptical incision, and circular incision, as shown in Figure 1. In this paper, the motion pair of the amplifying mechanism is mainly composed of the flexible hinge with a regular circular notch. It has high flexibility and a good rotation angle, which can better reduce the energy loss during the operation of the amplifying mechanism, increase the amplification factor of the lever amplifying mechanism, and improve the overall output performance of the platform [27], as shown in Figure 2. Compared with other kinds of flexible amplifying mechanisms, the biggest advantage of the system is that it can keep a kinematic linear relationship between input and output in principle [28,29,30,31]. By placing the PZT (Model P-820.2B) into the amplifier, the overall size of the amplifying mechanism becomes smaller, the structure tends to be more miniaturized, and part of the parasitic motion is reduced, as shown in Figure 3. In order to solve the error caused by the installation of the driver, the point contact between the piezoelectric ceramics and the driving part is kept. The parameters of PZT are shown in Table 1.

**Figure 4:**
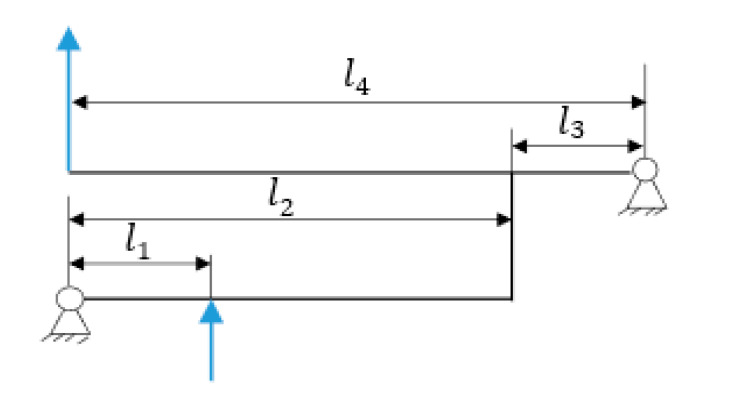
Schematic diagram of the lever amplification mechanism.

As for the compound lever amplification mechanism, as shown in Figure 4, *l*_1_ and *l*_2_ are defined as the input displacement and output displacement of the first-stage lever amplification, and the input displacement and output displacement of the second-stage amplifier are defined as *l*_3_ and *l*_4_, respectively.

The theoretical amplification ratio of the mechanism is calculated by the principle of the lever amplification mechanism [32]:

(1) $$A=\frac{l_{2}}{l_{1}}\cdot \frac{l_{4}}{l_{3}}$$

According to the data in Table 2, first-stage magnification ratio $\beta_{1}=\frac{18.5}{7}\approx 2.6428\;$; second-stage amplification ratio $\beta_{2}=\frac{25.5}{7}\approx 3.6428$. Substitute into Equation (1) to obtain the overall magnification ratio: $\beta =\beta_{1}\beta_{2}\approx 9.627$.

## 3. Finite Element Simulation

In order to verify the reliability of the theoretical calculation of the amplification ratio of the amplification mechanism, and the rationality of the related performance, the finite element simulation analysis is essential. Therefore, ANSYS Workbench software (ANSYS, Pittsburgh, PA, USA) was used to carry out the simulation analysis on the abovementioned amplification mechanism model. The ANSYS Workbench analysis process consists of four main steps: preliminary determination, pre-processing, loading and solving, and post-processing. The preliminary determination is the blueprint before the analysis, and the operational steps are the last three steps. The 3D modeling software, SolidWorks, was used to build the 3D model of the lever amplification mechanism and micro-operation platform, and the exported format was saved in Parasolid format. The model was then imported into ANSYS Workbench for static analysis. During the finite element simulation of the whole mechanism, the static platform of the mechanism was fixed, and then the required thrust or displacement was input, as shown in Figure 3, for simulation analysis.

### 3.1. Amplification Ratio

**Figure 5:**
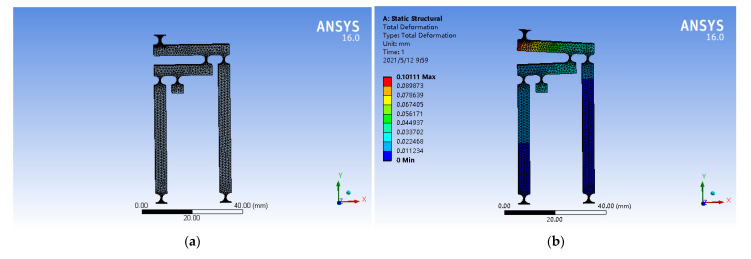
Meshing results of the mechanism (**a**); simulation of amplifier mechanism (**b**).

Aerospace aluminum alloy (AL)7075-T651 (Alcoa, Pittsburgh, PA, USA) was used as the material for the micro-operation platform. The model was opened in ANSYS Workbench, materials were added, and material parameters were set and modified, as shown in Table 3. In the grid size setting, the mesh refinement method included Proximity and Curvature. Smoothing was high, span angle center was fine, curvature normal angle was 18°, min size was 50 μm, and the rest were defaults. In principle, the finer the mesh, the higher the accuracy. However, considering the time and operation requirements of the computer calculation, we chose the appropriate meshing method. This is shown in Figure 5a [33].

ANSYS Workbench software showed that the output displacement is 101.11 μm when the input displacement is 10 μm. This is shown in Figure 5b.

**Figure 6:**
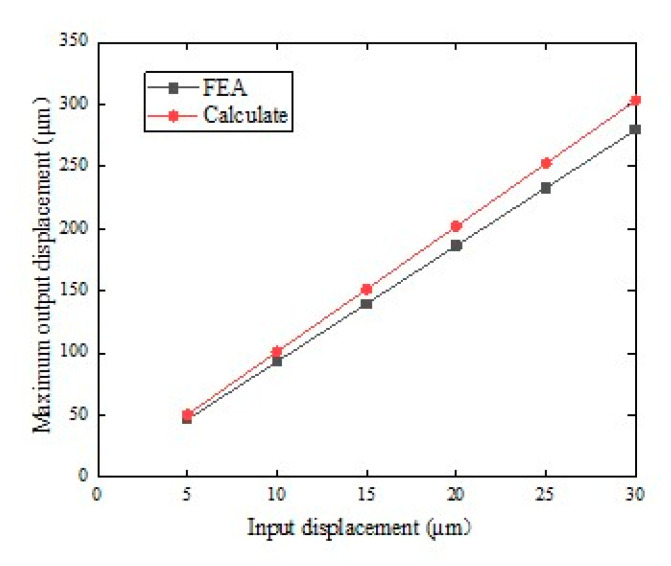
Relationship input displacement and maximum output displacement.

It can be known that the displacement range of PZT is 0–30 μm. In this paper, the displacement range was divided, and the step length was 5 μm. By applying a series of displacements of 5, 10, 15, 20, 25, and 30 μm to the input of the amplifying mechanism, a series of output displacements can be obtained. This is shown in Figure 6. Through finite element simulation, the simulation amplification ratio was about 10.111, and the theoretical value of A was 9.627. The error between the two was only 5.02%. The results show that the design of the amplifying mechanism is reasonable and reliable, and the theoretical calculation is accurate.

### 3.2. Input Stiffness

**Figure 7:**
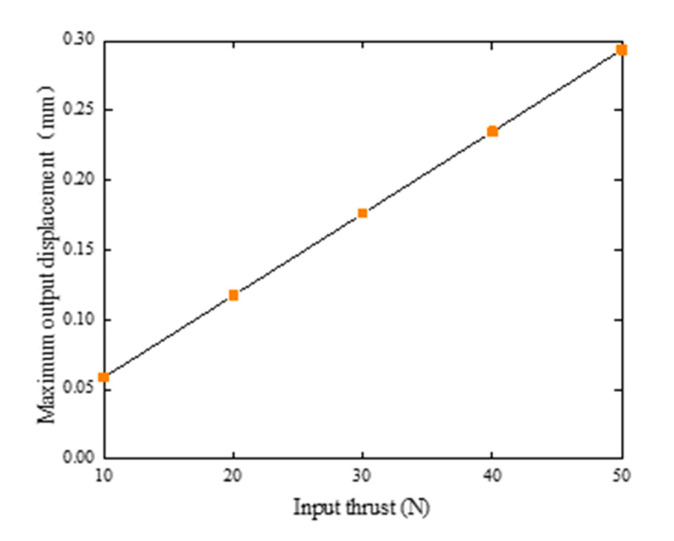
Relationship input thrust and maximum output displacement.

Parameters of the selected piezoelectric ceramic actuators were verified by the stiffness of the whole mechanism under various working conditions. It was determined whether the piezoelectric ceramic actuator meets the requirements of the output characteristics of the platform thus verifying the reliability of the platform. Under no-load conditions, the input ends of the three branch chains of the platform were loaded with thrusts of 10, 20, 30, 40, and 50 N, respectively. The relationship between the input thrust and the output displacement is shown in Figure 7. We can then figure out that the stiffness was 170.234 N/mm. The simulation results were lower than the actuators with the stiffness of 7000 N/mm (7 N/μm), which indicates that the platform can overcome the stiffness of the external mechanism and generate thrust. In addition, the flexibility of the platform determines the bearing capacity of the platform, which affects the positioning accuracy of the platform. In other words, the higher the stiffness, the higher the positioning accuracy [28].

### 3.3. Stress Analysis

**Figure 8:**
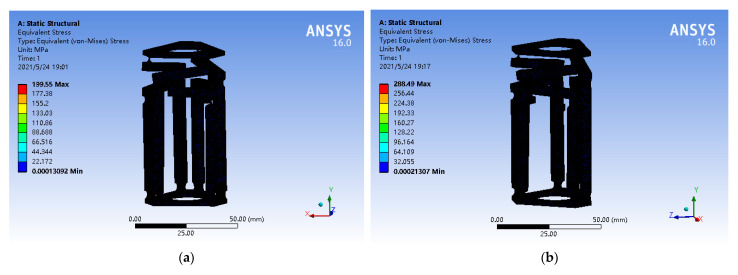
Static simulation of maximum stress value. (**a**) Three branches chain loaded displacement of 30 μm; (**b**) three branches chain loaded thrust of 50 N.

Under extreme conditions, it is considered whether the maximum stress is within the yield strength range of the selected AL7075-T6 aerospace aluminum alloy, with the maximum displacement of 30 μm and the maximum thrust of 50 N, respectively. The static simulation of the maximum stress value is shown in Figure 8. The maximum stress of the former was 199.55 MPa, while that of the latter was 288.49 MPa. The maximum stress value occurred at the circular curved hinge and was less than 503 MPa for the yield strength of AL7075-T6 aviation aluminum alloy. This ensures the stability of the mechanism.

### 3.4. Modal Analysis

Modal analysis by finite element analysis showed that the higher the natural frequency, the stronger the anti-interference ability of the micro-operation platform, and the faster the dynamic response. Table 4 shows the first six natural frequencies of the mechanism obtained by modal analysis. The natural frequency of the piezoelectric ceramic actuator P820.2B used in the mechanism is 15,000 Hz, which is greater than the natural frequency obtained by simulation. Therefore, the mechanism has better anti-interference ability, service life, and safety.

## 4. Kinematics Analysis

### 4.1. Kinematic Model

**Figure 9:**
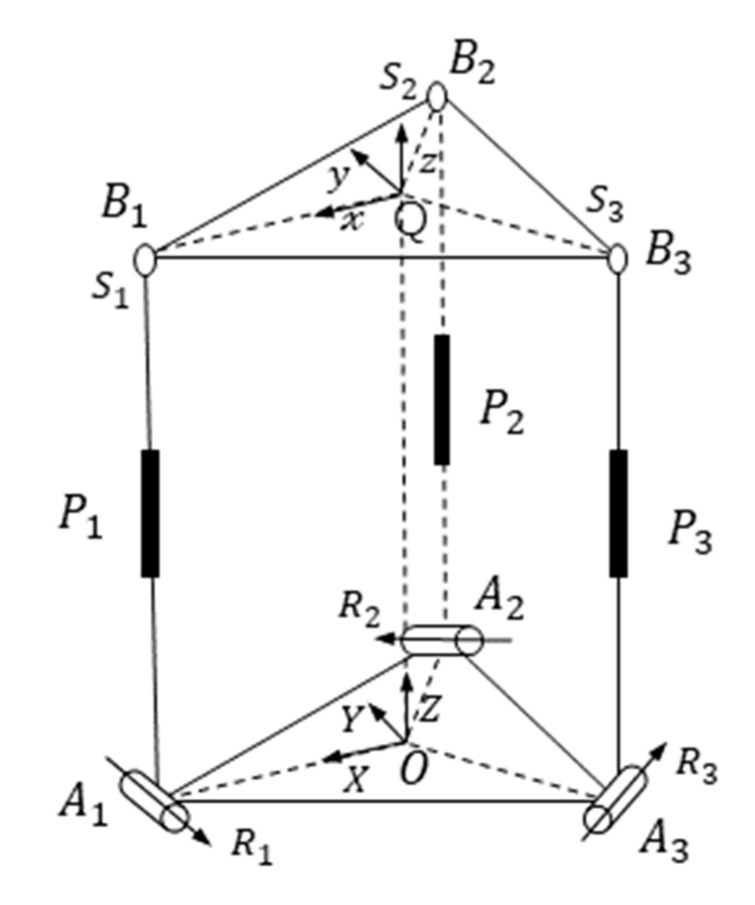
The 3-SPR kinematics model.

The 3-SPR (3 degrees of freedom consisting of a ball joint, a driving joint, and a revolute joint) flexible platform consists of a moving platform at the top, a branch chain in the middle, and a static platform at the bottom. The SPR branch chain is composed of a ball joint, a driving joint, and a revolute joint from top to bottom [34]. The three branches are symmetrically distributed. Figure 9 shows the 3-SPR kinematics model.

The static platform of the mechanism is simplified as vertices $A_{i}$, clockwise distribution and center O. The flexible rotating pairs at the upper and lower ends of the branch chain are $R_{i}$(i=1,2,3,4,5). The driving joint is $P_{i}$; the moving platform simplifies to vertices $B_{i}$, clockwise distribution and center Q. $OA_{i}=R$, $PB_{i}=r$, where *R*, *r* is the distance from the center to the vertex. The coordinate system O-XYZ is established at the center of the static platform at the point O, the positive crossing point $A_{i}$ of the X axis, the Y axis is parallel to $A_{3}A_{2}$, and the Z axis is perpendicular to the static platform. The moving coordinate system Q-xyz is established at the Q point of the moving platform. The cross point $B_{i}$ in the positive direction of the X axis, the Y axis is parallel to $B_{3}B_{2}$, and the Z axis is perpendicular to the moving platform.

### 4.2. Inverse Solution of Sports Degree Posture

In this paper, an analytical method was used to analyze the inverse solution of 3-SPR. $A_{i}$ in fixed coordinates:

(2) $$A_{1}^{A}=R\left[\begin{array}{c} 1\\ 0\\ 0 \end{array}\right],\;A_{2}^{A}=R\left[\begin{array}{c} -\frac{1}{2}\\ \frac{\sqrt{3}}{2}\\ 0 \end{array}\right],\;A_{3}^{A}=R\left[\begin{array}{c} -\frac{1}{2}\\ -\frac{\sqrt{3}}{2}\\ 0 \end{array}\right]$$

$B_{i}$ in moving coordinates:

(3) $$B_{1}^{B}=r\left[\begin{array}{c} 1\\ 0\\ 0 \end{array}\right],\;B_{2}^{B}=r\left[\begin{array}{c} -\frac{1}{2}\\ \frac{\sqrt{3}}{2}\\ 0 \end{array}\right],\;B_{3}^{B}=r\left[\begin{array}{c} -\frac{1}{2}\\ -\frac{\sqrt{3}}{2}\\ 0 \end{array}\right]$$

Definition $B_{i}^{A}$ is the coordinate representation of vertices in a fixed reference frame, $Q_{A}$ is the position vector of the center point *Q* of the moving platform in the fixed reference coordinate system *O*-*XYZ*, $R_{B}^{A}$ is the coordinate conversion moment of the moving platform relative to the static platform A, and the corresponding Euler angle is expressed by *xy*_1_*x*_2:_

(4) $$B_{i}^{A}=Q_{A}+R_{B}^{A}B_{i}^{B}$$

(5) $$R_{B}^{A}=\left[\begin{array}{ccc} x_{l} & y_{l} & z_{l}\\ x_{m} & y_{m} & z_{m}\\ x_{n} & y_{n} & z_{n} \end{array}\right]$$

(6) $$Q_{A}=\left[\begin{array}{c} x_{Q}\\ y_{Q}\\ z_{Q} \end{array}\right]$$

Substitute Equations (5) and (6) into Equation (4) to obtain:

(7) $$B_{i}^{A}=\left[\begin{array}{c} x_{Q}\\ y_{Q}\\ z_{Q} \end{array}\right]+\left[\begin{array}{ccc} x_{l} & y_{l} & z_{l}\\ x_{m} & y_{m} & z_{m}\\ x_{n} & y_{n} & z_{n} \end{array}\right]B_{i}^{B}$$

Substitute Equation (3) into Equation (7) to obtain:

(8) $$B_{1}^{A}=\left[\begin{array}{c} x_{Q}\\ y_{Q}\\ z_{Q} \end{array}\right]+\left[\begin{array}{ccc} x_{l} & y_{l} & z_{l}\\ x_{m} & y_{m} & z_{m}\\ x_{n} & y_{n} & z_{n} \end{array}\right]\left[\begin{array}{c} r\\ 0\\ 0 \end{array}\right]=\left[\begin{array}{c} x_{Q}+rx_{l}\\ y_{Q}+rx_{m}\\ z_{Q}+rx_{n} \end{array}\right]$$

(9) $$B_{2}^{A}=\left[\begin{array}{c} x_{Q}\\ y_{Q}\\ z_{Q} \end{array}\right]+\left[\begin{array}{ccc} x_{l} & y_{l} & z_{l}\\ x_{m} & y_{m} & z_{m}\\ x_{n} & y_{n} & z_{n} \end{array}\right]\left[\begin{array}{c} -\frac{1}{2}r\\ \frac{\sqrt{3}}{2}r\\ 0 \end{array}\right]=\left[\begin{array}{c} x_{Q}-\frac{1}{2}rx_{l}+\frac{\sqrt{3}}{2}ry_{l}\\ y_{Q}-\frac{1}{2}rx_{m}+\frac{\sqrt{3}}{2}ry_{m}\\ z_{Q}-\frac{1}{2}rx_{n}+\frac{\sqrt{3}}{2}ry_{n} \end{array}\right]$$

(10) $$B_{3}^{A}=\left[\begin{array}{c} x_{Q}\\ y_{Q}\\ z_{Q} \end{array}\right]+\left[\begin{array}{ccc} x_{l} & y_{l} & z_{l}\\ x_{m} & y_{m} & z_{m}\\ x_{n} & y_{n} & z_{n} \end{array}\right]\left[\begin{array}{c} -\frac{1}{2}r\\ -\frac{\sqrt{3}}{2}r\\ 0 \end{array}\right]=\left[\begin{array}{c} x_{Q}-\frac{1}{2}rx_{l}-\frac{\sqrt{3}}{2}ry_{l}\\ y_{Q}-\frac{1}{2}rx_{m}-\frac{\sqrt{3}}{2}ry_{m}\\ z_{Q}-\frac{1}{2}rx_{n}-\frac{\sqrt{3}}{2}ry_{n} \end{array}\right]$$

When the platform is perpendicular to surface $OQB_{i}A_{i}$, the following can be obtained:

(11) $$\left[\begin{array}{ccc} B_{i}^{A}(x) & B_{i}^{A}(y) & B_{i}^{A}(z)\\ A_{i}^{A}(x) & A_{i}^{A}(y) & A_{i}^{A}(z)\\ z_{l} & z_{m} & z_{n} \end{array}\right]=0(i=1,2,3)$$

It can be obtained from Equation (11):

(12) $$x_{n}z_{m}-x_{m}z_{n}=y_{n}z_{l}-y_{l}z_{n}$$

Because the displacement of the micro-moving platform is very small, the rotational angle is very small, so Formula (5) is substituted into Formula (12) to obtain:

(13) αβ(1−αγ)=βγ(1−αγ), α=γ

(14) $$\left[\begin{array}{ccc} x_{l} & y_{l} & z_{l}\\ x_{m} & y_{m} & z_{m}\\ x_{n} & y_{n} & z_{n} \end{array}\right]=\left[\begin{array}{ccc} 1 & \beta \gamma & \beta \\ \alpha \beta & 1-\alpha \gamma & -\gamma -\alpha \\ -\beta & \alpha +\gamma & -\alpha \gamma +1 \end{array}\right]$$

It can be obtained from Equations (12) and (14):

(15) $$y_{Q}=\frac{2z_{Q}\alpha +r((1-\alpha^{2})\alpha \beta -2\alpha \beta )}{\alpha^{2}-1}-2z_{Q}\alpha$$

(16) $$x_{Q}=\frac{2z_{Q}\beta +r(\beta^{2}+(1-\alpha \gamma ))-r({\left(1-\alpha \gamma \right)}^{2}+{\left(\alpha +\gamma \right)}^{2})}{2(1-\alpha \gamma )}\approx z_{Q}\beta$$

The length of the branch chain is $L_{i}$:

(17) $$L_{i}^{2}={\left|A_{i}^{A}-B_{i}^{A}\right|}^{2}$$

It can be concluded from (15) and (16) that:

(18) $$L_{i}=\sqrt{x_{Q}^{2}+y_{Q}^{2}+z_{Q}^{2}+R^{2}+r^{2}-2Rr+r(\sqrt{3}y_{Q}-x_{Q})+R{\left(x_{Q}-\sqrt{3}y_{Q}\right)}^{2}}$$

### 4.3. Sports Degree Posture Correct Solution

In this paper, an analytical method was used to analyze the positive pose solution of sports degree.

It can be obtained from Formula (18):

(19) $$L_{3}^{2}-L_{2}^{2}=2\sqrt{3}(R-r)y_{Q}$$

(20) $$\frac{L_{3}^{2}+L_{2}^{2}-2L_{1}^{2}}{2}=(3R+r)x_{Q}$$

It can be obtained from Equations (19) and (20):

(21) $$\frac{\beta }{\alpha }=\frac{\left(L_{3}^{2}+L_{2}^{2}-2L_{1}^{2})(R-r\right)}{2(L_{3}^{2}-L_{2}^{2})(3R+r)}$$

$a=\frac{\beta }{\alpha }$ is defined, then $\alpha =\frac{\beta }{a}$, according to Equations (15) and (16):

(22) $$L_{i}^{2}={\left(z_{Q}\beta \right)}^{2}+\frac{4}{a^{2}}{\left(z_{Q}\beta \right)}^{2}+z_{Q}^{2}+{\left(R-r\right)}^{2}-2Rz_{Q}\beta +2rz_{Q}\beta$$

(23) $$b=z_{Q}\beta =\frac{L_{3}^{2}+L_{2}^{2}-2L_{1}^{2}}{2(3R+r)}$$

Substitute (23) into (22) to obtain:

(24) $$z_{Q}=\sqrt{L_{i}^{2}-b^{2}-4\frac{b^{2}}{a^{2}}+2Rb-2rb-{\left(R-r\right)}^{2}}$$

According to Equations (19) and (20), we can get:

(25) $$\alpha =\frac{L_{3}^{2}-L_{2}^{2}}{-4\sqrt{3(R-r)}z_{Q}},\;\beta =\frac{L_{3}^{2}+L_{2}^{2}-2L_{1}^{2}}{2(3R+r)z_{Q}}$$

Since *R* and *r* are known, given the length of each branch chain link, the values of a and b can be obtained. Then, according to Equation (24), the value of $Z_{Q}\;$ can be obtained, thus the value of α and β can be obtained. Then, other parameters can be obtained by Formulas (15) and (16), and finally, the positive solution of the moving platform can be obtained. Equations (5)–(7) verify the forward kinematics solution.

### 4.4. Jacobian Matrix

This paper mainly studies a 3-DOF micro-operation platform, whose workspace is a three-dimensional space and has three driving joints; thus, its Jacobian matrix is a 3 × 3 partial derivative matrix. Assume that the 3-DOF motion direction of the platform is a rotation around *x* and *y* directions and translational motion is in the *z* direction. Let the velocity of the moving platform be $\dot{x}$, the input velocity be $\dot{y}$, and the Jacobian matrix J, then:

(26) $$\dot{x}=\left[\begin{array}{c} v\\ w \end{array}\right]$$

where $v={\left[\begin{array}{ccc} 0 & 0 & v_{Z} \end{array}\right]}^{T}$, $w={\left[\begin{array}{ccc} w_{x} & w_{y} & o \end{array}\right]}^{T}$, then:

(27) $$\dot{x}=\left[J(q)\right]\dot{q}$$

The generalized output velocity is divided into linear velocity v and angular velocity w. The formula above can be rewritten as:

(28) $$\dot{x}=\left[\begin{array}{c} v\\ w \end{array}\right]=\left[\begin{array}{c} v_{z}\\ w_{x}\\ w_{y} \end{array}\right]=\left[\begin{array}{ccc} J_{v1} & J_{v2} & J_{v3}\\ J_{w_{x}^{1}} & J_{w_{x}^{2}} & J_{w_{x}^{3}}\\ J_{w_{y}^{1}} & J_{w_{y}^{2}} & J_{w_{y}^{2}} \end{array}\right]\left[\begin{array}{c} {\dot{q}}_{1}\\ {\dot{q}}_{2}\\ {\dot{q}}_{3} \end{array}\right]$$

### 4.5. Workspace

**Figure 10:**
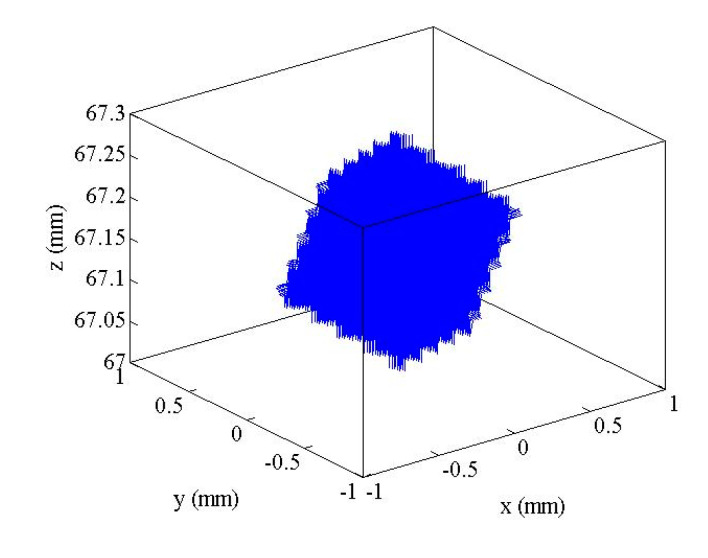
3-SPR workspace.

**Figure 11:**
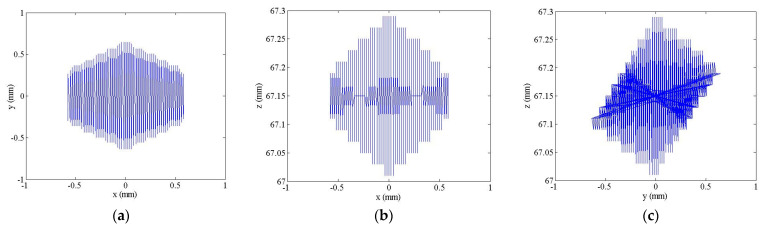
Plane workspace (**a**) *X*-*Y*; (**b**) *X*-*Z*; (**c**) *Y*-*Z*.

In this paper, Q point at the center of the moving platform was selected as the reference point. According to the formula derived from the inverse kinematics solution, there is a constraint relationship between the length of the branch chain and the coordinate position of Q point at the center of the moving platform. Given that the maximum stroke of the piezoelectric ceramic actuator was 30 μm, the expansion range of the branch chain was 0–303 μm after being driven by the actuator and the amplification mechanism. The space of 3-SPR micro-operation platform is shown in Figure 10. The corresponding workspace plane of the platform in the three directions is shown in Figure 11. The maximum movements of the platform in the three directions of the coordinate axis were 1160, 1280, and 303 μm, and the 3-SPR workspace was 1160 μm × 1280 μm × 303 μm. The platform has a large workspace, which can meet the requirements of large travel.

## 5. Conclusions

In this paper, a new type of flexible micro-motion platform is designed, which is driven by a piezoelectric ceramic actuator and amplified by a two-stage lever amplification mechanism. The static and modal analysis is carried out by using ANSYS software. The maximum stress in the ultimate state is obtained by loading the maximum displacement and the maximum thrust on the platform. The first six order natural frequencies of the mechanism were obtained by modal analysis on the micro-operation platform. The theoretical amplification ratio of the mechanism designed in this paper is 9.627, the simulation amplification ratio is 10.111, and the error between the theoretical amplification and simulation is 5.02%. In this paper, through the input stiffness, stress analysis, and modal analysis of the platform, the parameters and reliability of the platform are verified, and the stability and anti-interference ability of the mechanism are guaranteed. Finally, through the kinematics analysis, the space range of the platform is 1160 μm × 1280 μm × 303 μm, which can meet the requirements of large travel. However, due to the limitation of space, it cannot achieve larger motion magnification. Considering whether the flexible parallel micro-operation platform can be better applied to the manufacturing industry, and whether the relevant design of the platform is of great significance to the development of the discipline, we will conduct further experimental analysis on the platform to optimize the performance, which will be the next stage of work.

## Figures and Tables

**Table 1:** Performance parameters of P-820.2B stacked piezoelectric ceramic actuator.

Performance	Resolution Ratio	Displacement of 0 ~ 100 V	Thrust	Static Stiffness	Idling Frequency	Length	Capacitor
value	0.3 nm	0 ~ 30 μm	0 ~ 50 N	7 N/μm	15 kHz	44 mm	0.7 μF

**Table 2:** Design parameters of straight round flexure hinge.

Parameter	Value (mm)	Parameter	Value (mm)
*l*_1_	7	r	2
*l*_2_	18.5	t	0.5
*l*_3_	7	w	4
*l*_4_	25.5		

**Table 3:** Physical and mechanical parameters of the material Al7075-T651.

Parameter	Value	Unit
Young’s modulus	71.7	GPa
Tensile yield strength	503	MPa
Poisson’s ratio	0.33	—
Density	2810	kg/m^3^

**Table 4:** The first six natural frequencies.

Mode	Frequency (Hz)	Mode	Frequency (Hz)
1.	273.7	4.	504.13
2.	273.86	5.	609.59
3.	321.97	6.	610.57
